# Immune microenvironment heterogeneity of concurrent adenocarcinoma and squamous cell carcinoma in multiple primary lung cancers

**DOI:** 10.1038/s41698-024-00548-3

**Published:** 2024-02-29

**Authors:** Jiahao Zhang, Yiheng Huang, Yichao Han, Dong Dong, Yuqin Cao, Xiang Chen, Di Liu, Xueyan Cheng, Debin Sun, Hecheng Li, Yajie Zhang

**Affiliations:** 1 Department of Thoracic Surgery, Ruijin Hospital, Shanghai Jiao Tong University School of Medicine, 197 Rui Jin Er Road, Shanghai, 200025 China; 2 Genecast Biotechnology Co., Ltd., 88 Danshan Road, Xidong Chuangrong Building, Suite C 1310-1318, Xishan District, Wuxi City, Jiangsu 214104 China

**Keywords:** Non-small-cell lung cancer, Cancer microenvironment, Cancer genomics, Tumour immunology

## Abstract

The molecular profiles and tumor immune microenvironment (TIME) of multiple primary lung cancers (MPLCs) presenting as concurrent lung adenocarcinoma (ADC) and squamous cell carcinoma (SQCC) remain unknown. We aimed to clarify these factors. We performed whole-exome sequencing (WES), RNA sequencing (RNA-Seq), and multiplex immunohistochemistry (mIHC) for five patients with concurrent ADC and SQCC. We found the genetic mutations were similar between ADC and SQCC groups. RNA-Seq revealed that the gene expression and pathways enriched in ADC and SQCC groups were quite different. Gene set enrichment analysis (GSVA) showed that nine gene sets were significantly differentially expressed between the ADC and SQCC groups (*p* < 0.05), with four gene sets relevant to squamous cell features upregulated in the SQCC group and five gene sets upregulated in the ADC group. Reactome enrichment analysis of differentially expressed genes showed that the immune function-related pathways, including programmed cell death, innate immune system, interleukin-12 family signaling, and toll-like receptor 2/4 pathways, etc. were significantly enriched. Transcriptomic TIME analysis, with mIHC in patient specimens and in vivo validation, showed tumor-infiltrating immune cells were significantly more enriched and diverse in ADC, especially CD8 + T cells. Our results revealed that the transcriptomic profiles and TIME features were quite different between ADC and SQCC lesions. ADC lesions exhibited a more active TIME than SQCC lesions in MPLCs.

## Introduction

In recent years, owing to the increasing trend of population aging, the detection rate of multiple primary lung cancer (MPLC) is gradually increasing, accounting for 3–13% of the total number of lung cancer cases^1,2^. Compared with those of single primary lung cancer, differences exist in the biological characteristics, clinical manifestations, and treatment options for MPLCs^3^. Synchronous MPLCs (sMPLCs) are mostly pathologically identical and present as multiple primary adenocarcinomas (ADCs), whereas other sMPLCs present as pathologically different lung cancers. Reportedly, patients with different histological types account for approximately 3.8–61.5% of all cases of sMPLCs, most of which have concurrent lung ADC and squamous cell carcinoma (SQCC)^4^. Patients with sMPLCs with different histologic types had worse overall survival (hazard ratio [HR] = 10.00, *p* < 0.001) and recurrence-free survival (HR = 2.59, *p* = 0.023) than patients with MPLC with the same histologic type^4^. The same conclusion was also suggested previously^5,6^. Currently, there is no clear evidence regarding the reasons for this phenomenon, especially because of the lack of molecular biology and genetic analyses of these individual pathologically different sMPLC tumors.

Tumor immune microenvironment (TIME) has an important influence on tumor growth, immune escape, metastasis, therapeutic response, and patient survival^7,8^. Pathologically different sMPLCs feature distinct gene mutations, gene expression profiles, and intertumor TIME heterogeneity^9,10^. Wu^10^ reported two patients with sMPLCs who underwent surgical resection and were diagnosed with synchronous ADC and SQCC; they analyzed the genomic profiles of four lesions from these two patients. All four tumors showed different genomic profiles, suggesting a genetic multiplicity of origin. Most importantly, the TIME analysis showed different patterns in these four tumors, in which two ADC lesions presented more neoantigens, lacked HLA heterozygosity (HLA-LOH), and exhibited more CD8 + T-cell infiltration and cloning of T-cell receptor (TCRs), suggesting a more active TIME, than that of the two SQCC lesions.

However, except for a few case reports involving very limited research methods, TIME has rarely been studied directly in concurrent ADC and SQCC. In this study, we performed genomic, transcriptomic, and proteomic analyses of 10 sMPLC lesions from patients with concurrent ADC and SQCC using whole-exome sequencing (WES), RNA sequencing (RNA-Seq), and multiplex immunohistochemistry (mIHC). The aim of this multi-omics study was to investigate the differences in gene mutations, gene expression, and TIME between ADC and SQCC lesions in sMPLCs and to develop appropriate individualized treatment plans.

## Results

### Patient characteristics

**Fig. 1:**
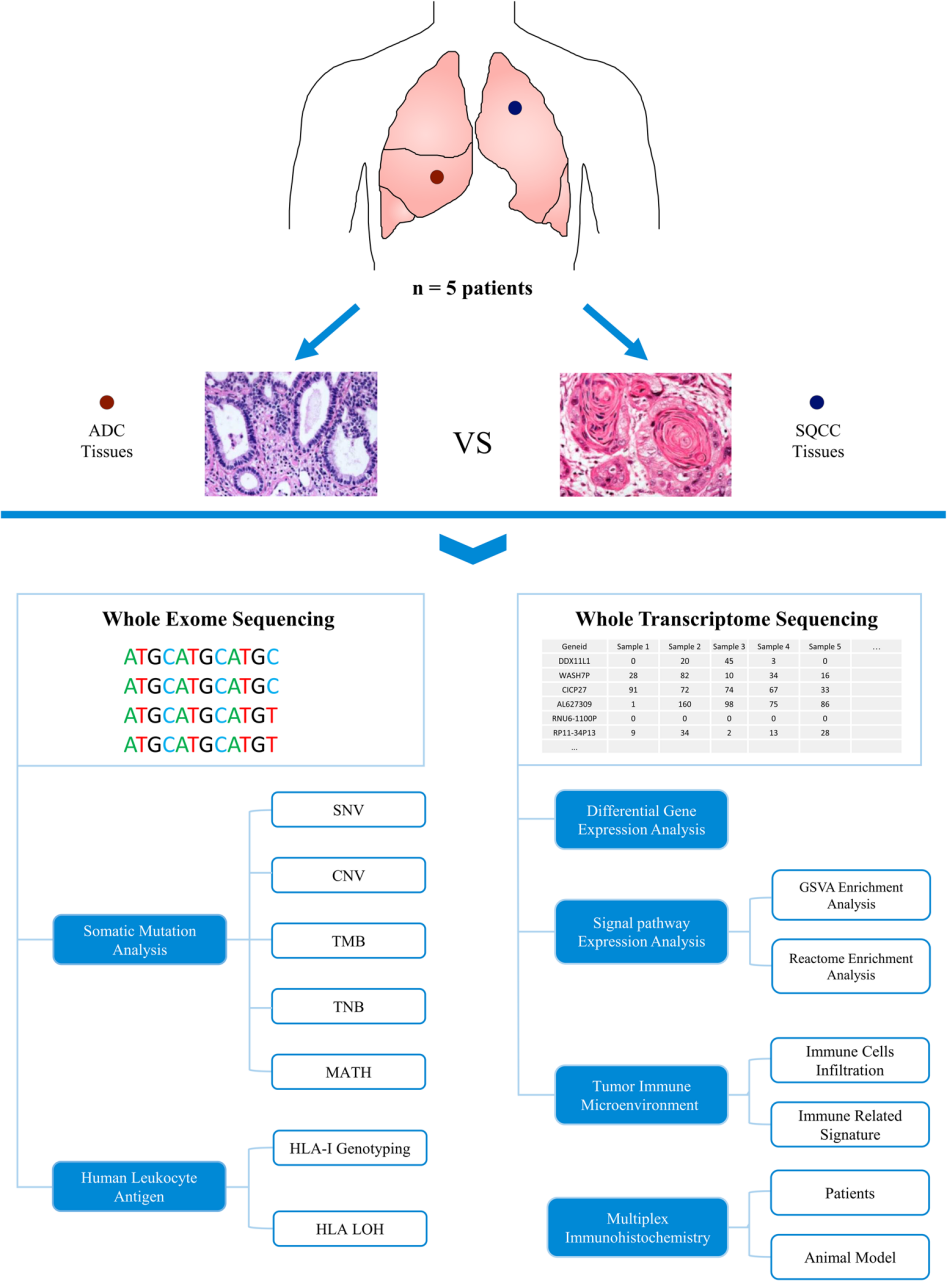
Flowchart of genomic and transcriptomic analyses of patients with concurrent ADC and SQCC. SNV single-nucleotide variant, CNV copy number variation, TMB tumor mutational burden, TNB tumor neoantigen burden, MATH mutant allele tumor heterogeneity, LOH loss of heterozygosity.

Five patients (four males and one female) with concurrent lung ADC and SQCC were included in the cohort. Figure 1 presents the flowchart of this study. The clinical and pathological characteristics of patients are summarized in Table 1. The median age was 68 (55–75) years, and all patients had a history of smoking. Three patients with ipsilateral lesions underwent simultaneous resection to remove all lesions, and the other two patients underwent a second resection to remove contralateral lesions. The median tumor diameter in the ADC group was 0.96 (0.5–2.5) cm and in the SQCC group was 2.5 (1.5–4.0) cm. The ADC group was staged as IA1 (three lesions), IA2 (one lesion), and IA3 (one lesion); the SQCC group was staged as IA2 (one lesion), IA3 (three lesions), and IB (one lesion). None of the five patients received postoperative adjuvant therapy. The median follow-up period of the cohort was 31 (range, 26–38) months, and no recurrence was found until June 2023.

**Table 1:** Clinical and pathological characteristics of five enrolled patients harboring both lung ADC and SQCC

Case, *n* = 5	Lesions, *n* = 10	Age(Y), sex	Ethnicity	Smoking history	Histology	Tumor size (cm), median = 1.75	Lymph node metastasis	Tumor location	Type of resection	Pathological stage	Date of surgery (DD/MM/YY)	Follow-up period (month), median = 31	Outcome
1	P1_AD	68, M	Chinese	Yes	Minimally invasive adenocarcinoma	0.6	No	LUL	Wedge resection	T1aN0M0, Stage IA1	02/03/2021	27	Alive without recurrence
	P1_SQ				Squamous cell carcinoma	4.0	No	RLL	Lobectomy	T2aNOM0, Stage lb	08/04/2021		
2	P2_AD	72, M	Chinese	Yes	Minimally invasive adenocarcinoma	0.6	No	RUL	Wedge resection	T1aN0M0, Stage IA1	06/08/2020	34	Alive without recurrence
	P2_SQ				Squamous cell carcinoma	2.2	No	RLL	Lobectomy	T1cN0M0, Stage IA3			
3	P3_AD	65, M	Chinese	Yes	Invasive adenocarcinoma	0.6	No	RLL	Lobectomy	T1aN0M0, Stage IA1	05/11/2020	31	Alive without recurrence
	P3_SQ				Squamous cell carcinoma	2.5	No	RLL + RML	Lobectomy	T1cN0M0, Stage IA3			
4	P4_AD	75, M	Chinese	Yes	Invasive adenocarcinoma	2.5	No	RUL	Lobectomy	T1cN0M0, Stage IA3	14/04/2021	26	Alive without recurrence
	P4_SQ				Squamous cell carcinoma	1.5	No	RUL	Lobectomy	T1bN0M0, Stage IA2			
5	P5_AD	55, F	Chinese	Yes	Minimally invasive adenocarcinoma	0.5	No	LUL	Wedge resection	T1aN0M0 Stage IA1	17/06/2021	38	Alive without recurrence
	P5_SQ				Squamous cell carcinoma	2.5	No	RUL	Lobectomy	T1cN0M0, Stage 1A3	07/04/2020		

*ADC* adenocarcinoma, *SQCC* squamous cell carcinoma, *LUL* left upper lobe, *RUL* right upper lobe, *RLL* right lower lobe, *RML* right middle lobe.

### Mutational profiles of concurrent ADC and SQCC

We first analyzed the mutational profiles of ADC and SQCC lesions. Somatic single-nucleotide variants (SNVs) and insertion/deletions (InDels) were the most common types of mutations.

**Fig. 2:**
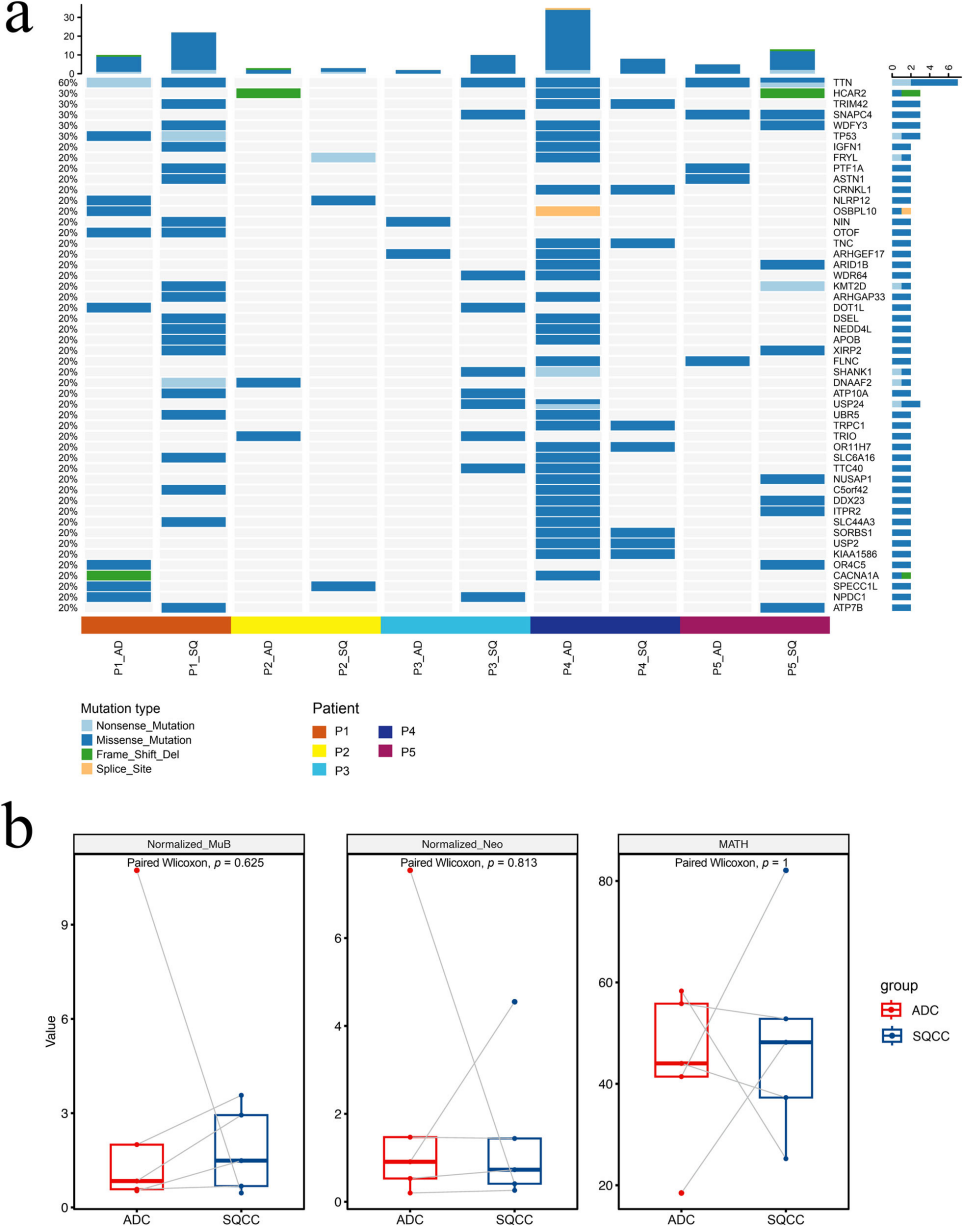
Analysis of mutation profiles and biomarkers of concurrent ADC and SQCC. **a** SNV and InDel landscape of adenocarcinoma and squamous cell carcinoma lesions. *Y* axis, mutational frequency of genes; scale bar on top, cumulative mutational number per column; scale bar on right, cumulative mutational number per row. **b** Differential analysis of TMB (MuB), TNB (Neo), and MATH between ADC and SQCC.

A comparison of SNVs and InDels between the ADC and SQCC groups (Fig. 2a) showed that the most frequent mutations (mutation frequency ≥30%) in all five patients were in *TTN* (60%), *HCAR2* (30%), *TRIM42* (30%), *SNAPC4* (30%), *WDFY3* (30%), and *TP53* (30%). Further comparative analyses of the population mutation frequencies of somatically mutated genes in the two groups showed no significant differences in mutated genes between the ADC and SQCC groups.

We also calculated TMB, TNB, and MATH in the ADC and SQCC groups and found no significant differences in TMB (*p* = 0.625), TNB (*p* = 0.813), and MATH (*p* = 1.000) between the ADC and SQCC lesions (Fig. 2b).

### HLA status of concurrent ADC and SQCC

**Fig. 3:**
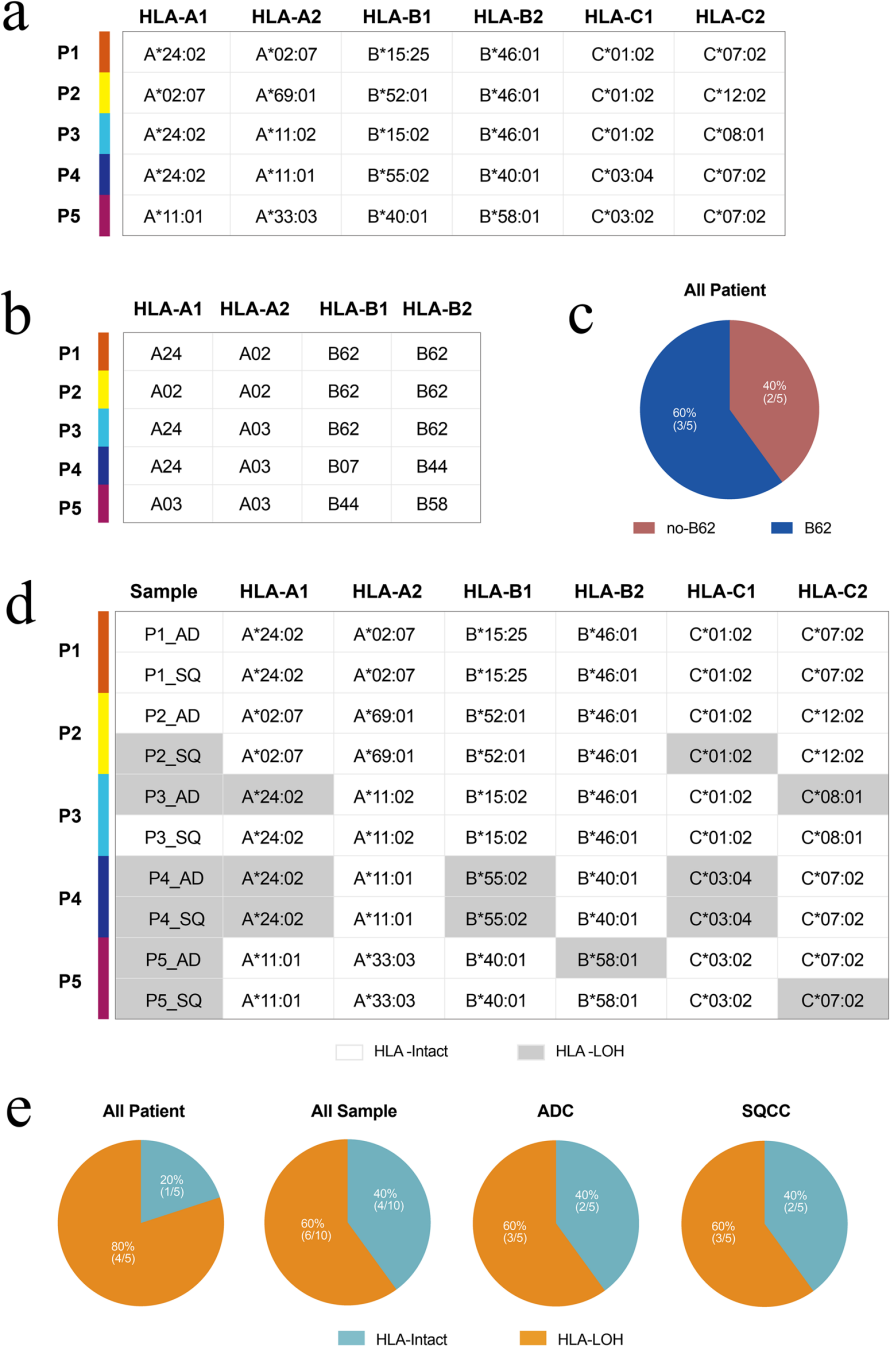
Analysis of HLA gene complex typing, HLA supertype, and HLA heterozygous deletions in concurrent ADC and SQCC lesions. **a** HLA genotype analysis of patients; **b** HLA hypertype analysis of patients; **c** Percentage of HLA-B62 hypertype occurrence in patients; **d** HLA heterozygous deletion occurrence in 7 of 10 lesions in total; and **e** Proportion of HLA-LOH in all samples, all patients, and two groups.

We also performed typing of the five patients’ HLA gene complex and HLA supertype analysis using the HLA-HD software based on WES (Fig. 3a–e). The results showed that all five patients were heterozygous for the HLA genotype (Fig. 3a), and three of these five patients (60%, P1, P2, and P3) carried the HLA-I B62 supertype (Fig. 3b, c). Given the impact of HLA polymorphisms on sequence alignment and copy number detection, loss of HLA heterozygosity (HLA-LOH) analyses were performed for all 10 lesions. The results showed that HLA-LOH was present in six lesions (6/10, 60%) from four of five (80%) patients (Fig. 3d; P2, P3, P4, and P5 marked in gray). HLA-LOH occurred in three lesions (3/5, 60%) in the ADC group and in three lesions (3/5, 60%) in the SQCC group (Fig. 3e).

### Transcriptomic features of concurrent ADC and SQCC

**Fig. 4:**
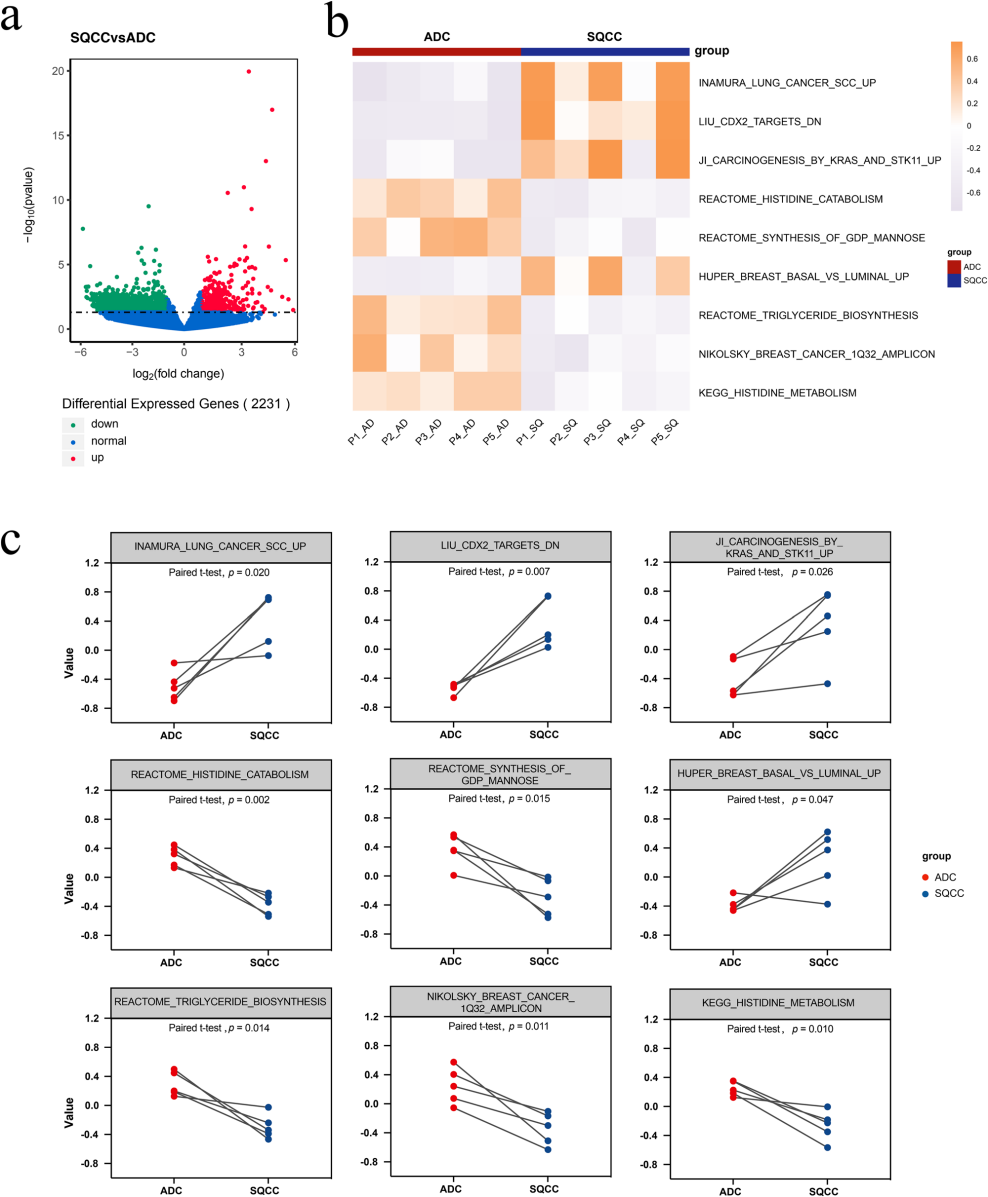
Gene expression analysis in MPLC ADC and SQCC lesions. **a** Volcano plot of up- and downregulated genes in SQCC compared with ADC, red: genes upregulated in SQCC, green: genes downregulated in SQCC; **b** Heatmap of differently expressed C2 gene sets between ADC and SQCC, color scale on right: z score; **c** Paired scatter plot of differently expressed C2 gene sets between ADC and SQCC.

Subsequently, we analyzed the transcriptomic features of ADC and SQCC lesions. Gene counts were generated for each sample and collated into a matrix for a paired regression analysis using DESeq2. We found that the genes in the ADC and SQCC groups were highly differentially expressed, with the number of differentially expressed genes reaching 2231, in which 544 genes were upregulated, and 1687 genes downregulated in SQCC (Fig. 4a).

GSVA on the whole gene set was then conducted using the molecular signature database (MsigDB). Analysis of the C2 curated gene sets revealed (Fig. 4b, c) that nine gene sets (7233 gene sets in total) were found to be significantly different (*p* value < 0.05) between the SQCC and ADC groups. Four gene sets were upregulated in SQCC, including INAMURA_LUNG_CANCER_SCC_UP (SQCC-associated signature gene, *p* = 0.020), LIU_CDX2_ TARGETS_DN (gene downregulated in HET1A esophageal epithelial cells, *p* = 0.007), JI_CARCINOGENESIS_BY_KRAS_AND_STK11_UP (gene upregulated in KRAS activation and STK11 deletion-driven primary lung tumors, *p* = 0.026), and HUPER_BREAST_BASAL_VS_LUMINAL_UP (genes upregulated in basal mammary epithelial cells, *p* = 0.047). The five gene sets significantly upregulated in the ADC group were REACTOME_HISTIDINE_CATABOLISM (histidine catabolism, *p* = 0.002), REACTOME_ SYNTHESIS_OF_GDP_MANNOSE (mannose synthesis, *p* = 0.015), REACTOME_TRIGLYCERIDE_BIOSYNTHESIS (triglyceride biosynthesis, *p* = 0.014), NIKOLSKY_BREAST_CANCER_1Q32_AMPLICON (genes within amplicon 1q32 identified in breast tumor samples, *p* = 0.011), and KEGG_HISTIDINE_METABOLISM (histidine metabolic pathway, *p* = 0.010).

### Enrichment analysis of DEGs between concurrent ADC and SQCC

**Fig. 5:**
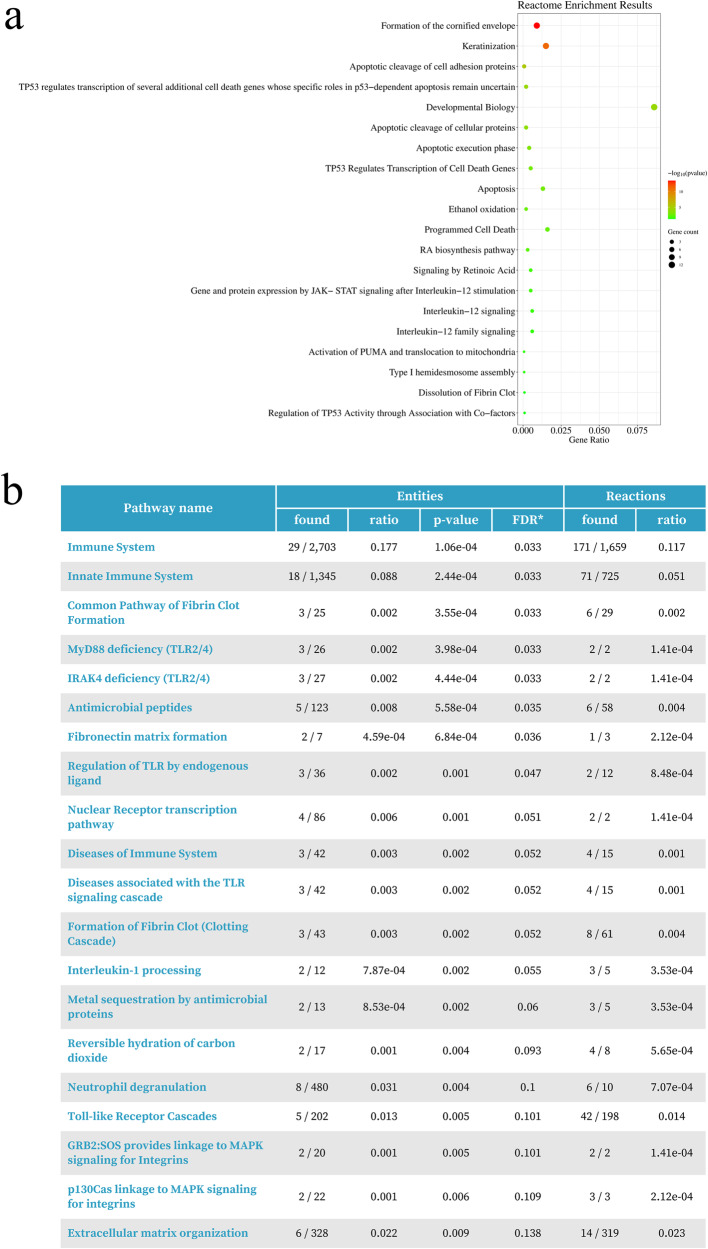
Enrichment analysis of differentially expressed genes between ADC and SQCC groups. **a** Bubble chart of top 20 enriched pathways determined using Reactome database analysis, sorted by lowest *p* values; **b** Top 20 enriched pathways from immune system cluster in the Reactome event hierarchy, sorted by lowest *p* values.

A subsequent enrichment analysis of all 2231 DEGs was performed using the Reactome database^11^. The top 20 pathways with the lowest *p* values from the results are shown in Fig. 5a, of which, programed cell death (*p* = 0.003), IL-12 signaling (*p* = 0.019), and IL-12 family pathways (*p* = 0.024) were significantly enriched, which suggests that the expression of immune-related pathway components may differ between the two groups.

Therefore, to explore potential differences in immune function pathways between ADC and SQCC, we focused on the immune system clusters of the Reactome event hierarchy^11^. The top 20 enriched pathways (with *p* < 0.01) in this cluster are shown in Fig. 5b, and the top five pathways included the immune system, innate immune system, common pathway of fibrin clot formation, MyD88 deficiency (TLR2/4), and IRAK4 deficiency (TLR2/4). Taken together, these results may indicate a potential difference in immune function between the two groups.

### TIME analysis of concurrent ADC and SQCC

**Fig. 6:**
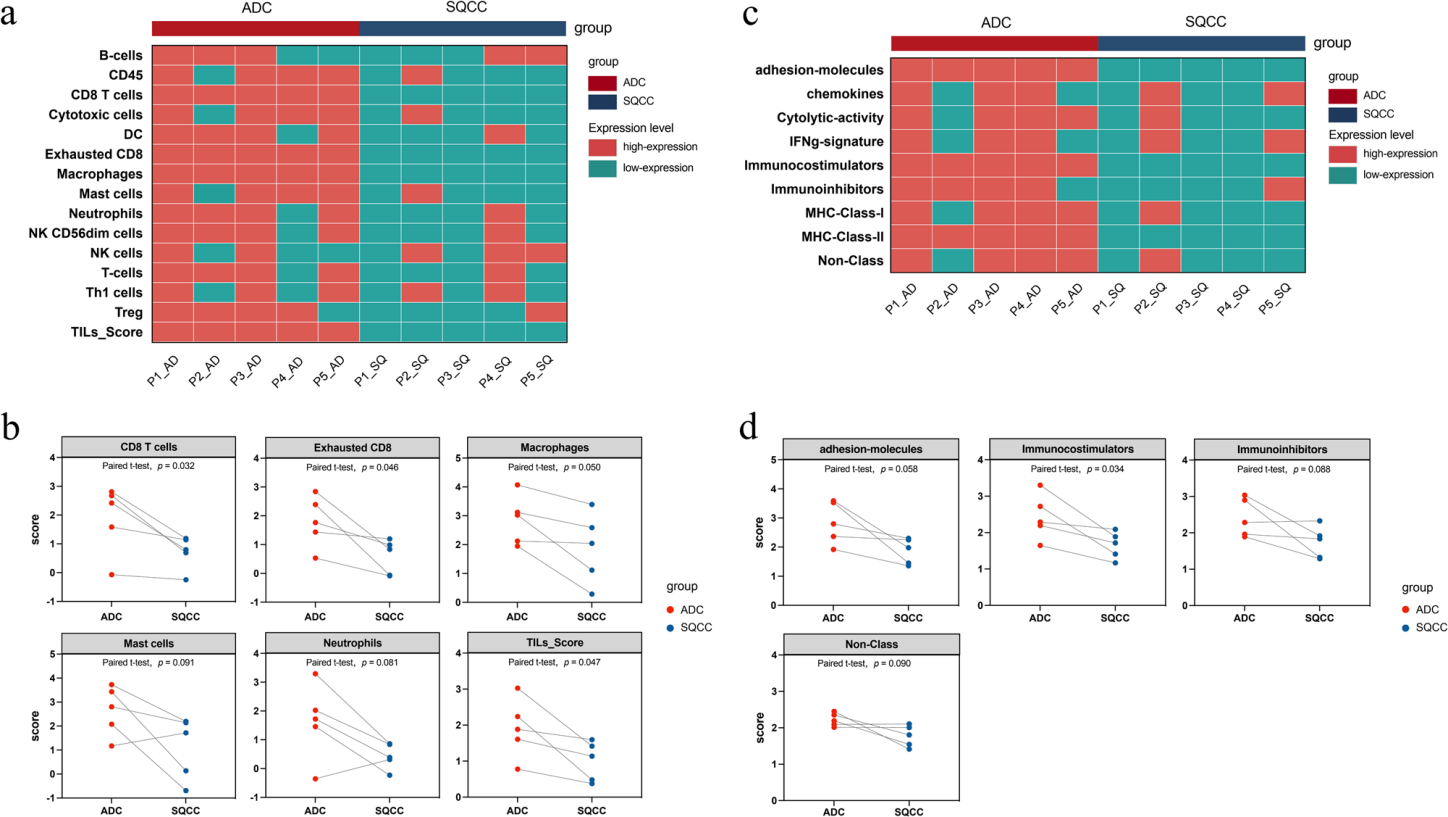
Analysis of the tumor immune microenvironment between ADC and SQCC. **a**, **b** Differential analysis of the abundance of infiltrating immune cell populations between groups based on the co-expression dataset. **a** Double heatmap and **b** paired scatter plot. **c**, **d** Comparative analysis of immune characteristics between the two groups. **c** Double heatmap and **d** paired scatter plot. High-expression, the relative expression level of gene sets in one lesion is higher than that in the histologically paired lesion (e.g., P1_AD and P1_SQ).

TIME plays an important role in the immunotherapy^12^. To evaluate the TIME discrepancy between the two groups, we analyzed the differences in the TIME of ADC and SQCC regarding the spectrum of infiltrating immune cells and molecules and summarized the results in Fig. 6.

TILs have been shown to be important predictive and prognostic biomarkers of tumors and are closely associated with tumor clinical outcomes^7^. A comparison of the abundance of the 14 TIL populations using the co-expression method revealed that the abundance of immune cell populations (TIL score) was significantly higher in the ADC group than in the SQCC group (Fig. 6b, *p* = 0.047). A paired comparative analysis between the groups revealed that CD8 + T cells (*p* = 0.032), exhausted CD8 + T cells (*p* = 0.045), and macrophages (*p* = 0.050) infiltrated significantly more into tumor samples in the ADC group than in the SQCC group. The number of neutrophils (*p* = 0.061) and mast cells (*p* = 0.091) in the ADC group tended to be higher than those in the SQCC group; however, the difference was not statistically significant (Fig. 6a, b).

The immune microenvironment characteristics of the two groups were further analyzed, and the RNA signature scores were calculated (Fig. 6c, d). Comparative analyses revealed that the immune characteristics that were significantly different between the SQCC and ADC groups comprised immunocostimulators, which were significantly more abundant in ADCs than in SQCCs (*p* = 0.034). Moreover, there was a tendency for the adhesion molecule (*p* = 0.058), immunoinhibitors (*p* = 0.088), and non-class (*p* = 0.090) scores to be higher in the ADC group than in the SQCC group; however, the difference was not significant (Fig. 6d). We also analyzed the expression of markers and suppressor genes associated with exhausted CD8 + T cells, including PD-1, PD-L1, and CD244, between SQCC and ADC lesions (Figure Supplementary 2) and found no significant difference in PD-1, PD-L1, and CD244 expression between both groups (*p* > 0.05).

**Fig. 7:**
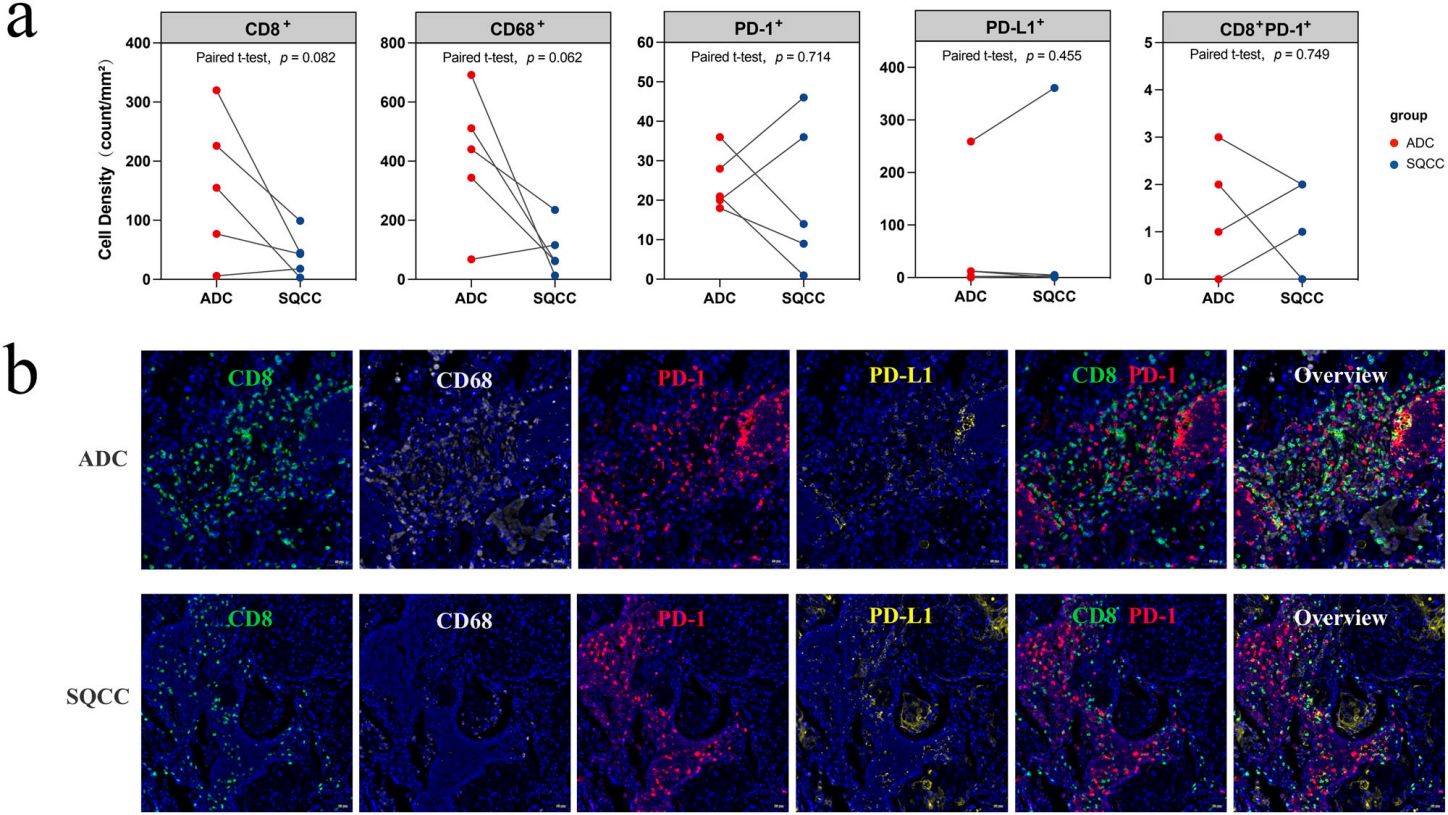
Validation of TIME characteristics using multiplex immunohistochemical (mIHC) analysis of concurrent ADC and SQCC. **a** Paired scatter plot of immune cell density in tumor between ADC and SQCC groups; **b** Representative costaining immunofluorescence images of ADC and SQCC lesions from one patient (×20 magnification), labeled with DAPI (blue), CD8 (green), CD68 (gray), PD-1 (red), PD-L1 (yellow), scanned using the TissueFAXS SL imaging system.

### Proteomic validation of TIME characteristics

Next, we validated our findings from the transcriptomic analysis at the proteomic level. mIHC was applied on all 10 samples to characterize the composition of the TIME. Expression of CD8, CD68, PD-1, PD-L1, and PANCK was detected using immunofluorescence and quantitively analyzed. The cell density of CD68 in tumor showed a clear tendency to be significantly higher in ADC than SQCC (Fig. 7a, *p* = 0.062). Cytotoxic CD8 + T cells in TIME are critical for recognizing and killing tumor cells during immunotherapy^12^. We also found a notable trend towards a significant increase in the cell density of CD8 (Fig. 7a, *p* = 0.082) in ADC. The representative images (Fig. 7b) of mIHC also demonstrated a higher density of CD8 and CD68 in ADC than in SQCC. Taken together, these results indicate that the CD8 and CD68 cell density clearly tends to be higher in ADC than in SQCC in MPLCs, which corroborates our findings of the transcriptomic analysis.

**Fig. 8:**
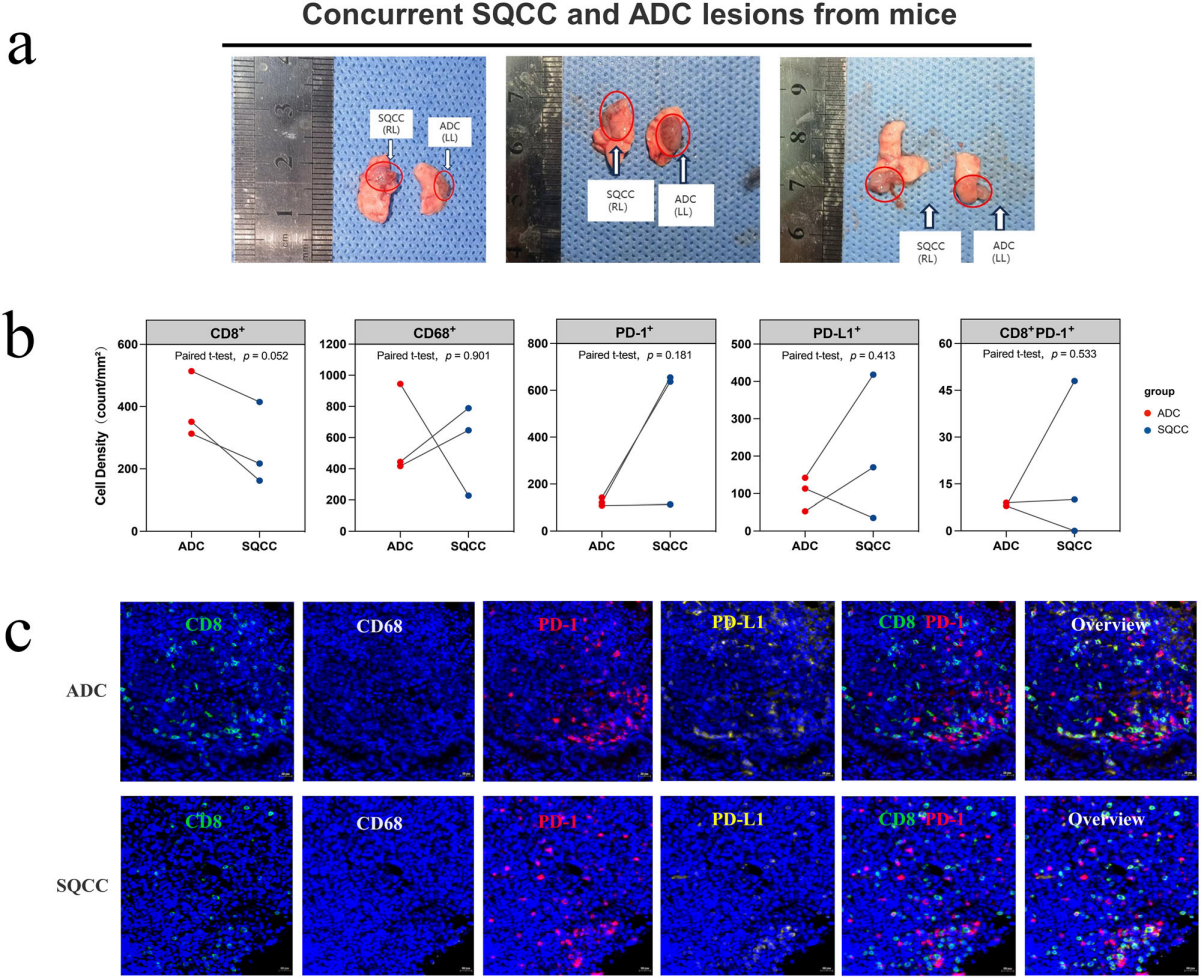
In vivo mIHC validation of TIME characteristics in MPLC animal model. **a** Photographs of simultaneous bilateral KLN-205 and LLC cancer cell in-situ injection surgery for animal model construction and concurrent SQCC and ADC tumor pairs included for analysis from three mice; **b** Paired scatter plot of immune cell density in tumor between ADC and SQCC groups from mIHC results of mice; **c** Representative mIHC images of ADC and SQCC lesions from one mouse (×20 magnification), labeled with DAPI (blue), CD8 (green), CD68 (gray), PD-1 (red), and PD-L1 (yellow), scanned using the TissueFAXS SL imaging system. RL, right lung; LL, left lung.

In the animal model, we observed successful growth of ADC and SQCC lesions after 10 days of tumor implantation surgery with KLN-205 cells (SQCC cell line, right lung) and LLC cells (ADC cell line, left lung). We collected three pairs of concurrent ADC and SQCC lesions from the mice (Fig. 8a) and performed mIHC analyses to determine the expression levels of CD8, CD68, PD-1, PD-L1, and PANCK on these lesions. We found that the CD8 cell density was significantly higher in ADC lesions than in SQCC lesions (Fig. 8b, c, *p* = 0.052), which is consistent with the findings from the transcriptomic and proteomic analyses in these patients.

## Discussion

TIME has been the hotspot of research in MPLC, and some research progress has been made in multiple primary lung ADCs^13^. Currently, TIME has rarely been studied directly in concurrent ADC and SQCC, with only a few case reports involving limited research methods^10^. Our multi-omics study of pathologically different sMPLC lesions and exploration of their TIME characteristics assist in understanding the mechanism of their progression and potential differential therapeutic responses.

In this study, we compared the gene mutation profiles of ADC and SQCC lesions in MPLCs. We confirmed the similarity in genetic mutations between both groups and found *TTN* was the most frequently mutated gene. In previous studies, *TTN*-mutated lung ADC had an inflammatory TIME characterized by enrichment of activated immune cells and elevated immune scores^14^. Moreover, we found that the HLA-I B62 supertype and HLA-LOH were frequent in these patients. According to previous studies, HLA-LOH is one of the main mechanisms of immune escape in lung cancer, leading to an impaired therapeutic response and poor survival^15,16^.

In GSVA analysis of the whole transcriptome, we found that four gene sets related to squamous cell features were significantly upregulated in SQCC, and five gene sets were downregulated in SQCC, of which four were metabolism-related gene sets. Among them, the downregulation of both mannose metabolism and histidine catabolism pathways may contribute to the potential lower sensitivity of SQCC to chemotherapy or immunotherapy than that of ADC, according to previous research data^17–19^. In the Reactome enrichment analysis of DEGs, we observed potential differences in the immune function, including the IL-12 family signaling pathways and TLR2/4 pathway between ADC and SQCC. According to previous studies, the IL-12 pathway activates multiple immune cells and stimulates the production of cytokines to enhance the cell-mediated immune response against tumor cells in the TIME^20^. TLR2/4 pathway has been reported to influence chronic inflammation in tumors, activates dendritic cell traffic and its associated tumor-specific, cytotoxic T-cell responses in the TIME^21^, and reduces tumor progression through multiple mechanisms^22^,^23^. These findings aroused our interest in TIME differences between the two groups.

Then, the transcriptomic microenvironment analysis found a more active TIME in ADC than in SQCC, with significantly more CD8 + T cells, exhausted CD8 + T cells, macrophage infiltration, and immunocostimulators expression. To validate our findings from the transcriptomic analysis, we performed a proteomic analysis with mIHC on specimens from both patients and the animal model, which also indicated higher infiltration of TILs in ADC than in SQCC in MPLC lesions, especially of CD8 + T cells. These findings supported the different patterns of TIME features in concurrent ADC and SQCC. In Wu’s case report^10^, similar features of the TIME were also found in two ADC lesions, namely, more neoantigens, more infiltrating CD8 + T cells, and more clonal TCRs, than in the other two SQCC lesions. Our study enrolled a larger cohort and further depicted the TIME features of concurrent ADC and SQCC lesions using more abundant transcriptomic analyses, and we validated our results with mIHC on patients and the animal model.

However, it is worth noting that our finding of TIME features in pathologically different MPLCs may be different from those of solitary ADC or SQCC. Qu Y^24^ analyzed the TIME of ADC and SQCC with gene expression data from The Cancer Genome Atlas and found that the numbers of Tregs and M2 macrophages were higher in ADCs, whereas those of activated CD4 + T cells and M1 macrophages were higher in SQCC. CD8 + T cells exhibited no significant difference between ADC and SQCC. Wang C^25^ collected 10 solitary ADC and 9 solitary SQCC lesions from 19 patients with non-small cell lung cancer (NSCLC), and the single-cell RNA sequencing (scRNA-seq) and mIHC analysis suggested that CD8 + T cells (*p* = 0.069), B cells (*p* = 0.020), Tregs (*p* = 0.007), and mast cells (*p* = 0.032) were more abundant in SQCC than in ADC. In contrast, we found a higher infiltration of TILs in ADC than in SQCC in concurrent lesions. This finding suggests the potential inconsistency of the TIME between MPLCs and solitary NSCLC, which may result in different treatment responses, especially when applying immunotherapy, and more studies are needed to develop individualized treatment modalities.

The potential advantage of this study is that although the prevalence of concurrent lung ADC and SQCC is rather low, we enrolled a cohort of 5 patients and 10 lesions and performed a comprehensive multi-omics study on all lesions simultaneously. Because each patient in this cohort had concurrent ADC and SQCC, ADC and SQCC lesions with the same genetic background were paired, which, to a certain extent, excluded the influence of a genetic background discrepancy between patients on the analysis results. Moreover, our study was conducted on multi-omics levels, which provided a relatively complete and reliable description of the difference in the TIME between concurrent ADC and SQCC lesions at genomic, transcriptomic, and proteomic levels.

This study has some potential limitations. First, the relatively small number of patients included in the study is not representative of all MPLC patients with pathologically different lesions. A larger sample size for validation is required. In this study, a transcriptome analysis was performed using bulk RNA-Seq, which does not provide detailed transcriptomic information on cell subpopulations, cell status, and intra-neoplastic heterogeneity of MPLC lesions. The TIME of these lesions should be more carefully investigated in the future using scRNA-seq or spatial transcriptomics. Besides, long-term follow-ups for survival and treatment data of these patients are needed.

In conclusion, to our knowledge, our study is the first to include a cohort of five sMPLC patients with concurrent ADC and SQCC for genomic transcriptomic and mIHC research. By analyzing the genomic profiles of 10 lesions in total, we found that, although the mutational profiles of ADC and SQCC were similar, the transcriptional profiles were significantly different. The TIME analysis using transcriptomic data and proteomic mIHC validation for both patients and the animal model indicated that the tumor-infiltrating immune cells and immune molecules were significantly different. The TIME of ADC lesions was significantly more active than that of SQCC lesions, especially with more infiltration of CD8 + T cells. Our findings indicate the potential inconsistency of the TIME between MPLCs and solitary NSCLC, and individualized treatment modalities may be needed for MPLC patients.

## Methods

### Patients and specimens

We enrolled five patients with MPLC harboring concurrent ADC and SQCC who underwent surgical resection between January 2020 and December 2021 in the Department of Thoracic Surgery, Ruijin Hospital, Shanghai. Pathologists diagnosed all included patients according to the 2015 World Health Organization classification^26^. Clinical information such as age, sex, tumor-node-metastasis classification, and stage was collected and analyzed.

Our study complied with the ethical standards of the Helsinki Declaration. This study was approved by the Ethics Committee of Ruijin Hospital, affiliated with Shanghai Jiao Tong University School of Medicine, and the number of ethics approvals is ‘RJ-2021-376’. All patients signed an informed consent form before enrollment and tissue donation.

### Sample preparation and DNA sequencing

The samples were compliant with the ‘Guidance of the Ministry of Science and Technology (MOST) for the Review and Approval of Human Genetic Resources’. All specimens, including tumor and matched controls, were fixed using paraformaldehyde within 24 h after surgical resection, followed by dehydration and embedding in paraffin. Prior to nucleic acid extraction, the specimens were sectioned and deparaffinized. Genomic DNA was then extracted from formalin-fixed paraffin-embedded (FFPE) tissues using the MagPure FFPE DNA kit (MGBio Tech, Shanghai, China). It was fragmented to an average size of 180–280 bp using a Covaris S220 sonicator (Genecast Biotechnology Co., Ltd., Wuxi, China). Libraries were constructed using Agilent Sure Select Human All Exon V6 for exome sequencing according to the manufacturer’s protocol and subsequently sequenced on Illumina NovaSeq 6000 (Novogene Corp., Sacramento, CA) for generating paired-end 300 bp reads.

The raw data were quality-controlled via fastp^27^, and qualified reads were aligned to the human reference genome (build hg19) using the BWA-MEM^28^ aligner. Alignments were sorted, and duplications were removed using Samtools^29^ (http://samtools.sourceforge.net) and Picard (https://broadinstitute.github.io/picard/), respectively. BAM files were realigned, and base quality scores were recalibrated using GATK (https://gatk.broadinstitute.org/hc/en-us). MuTect2 (http://www.broadinstitute.org/cancer/cga/mutect) was used to identify somatic SNVs and small insertions or deletions with default parameters based on paired alignment files (tumor and matched germline). Mutations were excluded if the following criteria were not met: (1) non-silent variants, including missense, nonsense, frameshift, and splice-site variants; (2) high-confidence variants with variant allele frequencies >0.03; (3) depth ≥50×; (4) rare variants with frequencies <0.01 in databases (ExAC^30^ and gnomAD^31^). Copy number variations (CNVs) were paired-called using the CNVkit software with a copy number threshold of 4 for CNV gain and 1.2 for CNV loss^32^.

### Tumor mutational burden (TMB), tumor neoantigen burden (TNB), and mutant allele tumor heterogeneity (MATH) Calculation

To determine the TMB, the absolute mutation counts of the tumor samples against the mutation spots of the normal samples were calculated using the following formula: absolute mutation counts × 1,000,000/exonic base number and measured in mutations per Mb. TNB was calculated as the number of neoantigens per Mb in the genomic region using pVACseq. MATH was calculated as ×100 median absolute deviation/median variant allele frequency.

### HLA-I typing and loss of heterozygosity

We analyzed the raw WES data of matched normal and tumor samples using the algorithm LOH-HLA^15^. For each patient, the 4-digit HLA type was inferred using OptiType^33^, which uses a normal germline BAM file as the input. A loss of heterozygosity was reported when a copy number <0.5 was estimated using binning and B-allele frequency settings and when the significance of allelic imbalance was reached (*p* < 0.01), while counting each sequencing read once.

### RNA sequencing

Total RNA was extracted from the FFPE samples using the RNeasy FFPE Kit (QIAGEN USA, Germantown, MD, USA) according to the manufacturer’s instructions. Total RNA (50 ng) was used for RNA library construction using SMARTer Stranded Total RNA-Seq Kit v2 (Takara Bio USA, Mountain View, CA, USA). The cDNA libraries were sequenced on Illumina NovaSeq 6000 for generating paired-end 300 bp reads.

### Gene expression analysis

After quality control, clean data were aligned to the reference genome (GRCh37) with STAR (version 2.7.8a)^34^. FeatureCounts^35^ was used to estimate the expression level of each gene. Gene expression was quantified as transcripts per million (TPM). We used the DESeq2^36^ package of R software (4.2.0) to screen differentially expressed genes between comparisons, with the following threshold: |log2(Fold Change)|>1 and *p* value < 0.05. Paired regression analysis using DESeq2 was performed to identify differentially expressed genes (DEGs) associated with histologically paired lesions. Gene set enrichment analysis (GSVA) (https://www.bioconductor.org/packages/release/bioc/html/GSVA.html)^37^ of whole gene sets was conducted using the canonical Broad C2 collection of gene sets in the molecular signature database (MsigDB) based on the expression data.

### Enrichment analysis of DEGs

A subsequent pathway enrichment analysis using the list of DEGs was performed using the Reactome database (https://reactome.org/)^11^. To better understand the differences in immune function, the clusters of immune system in the Reactome event hierarchy were extracted and analyzed.

### Scoring of tumor-infiltrating lymphocytes

We used signature genes to measure immune cell subpopulations in the tumor microenvironment. The immune cell scores, including tumor-infiltrating macrophage, T-cell, dendritic cell, Regulatory T-cell, and mast cell scores^38,39^ represent a convenient technique for extracting detailed information about the tumor immune contexture in samples. Given their association with clinical response to checkpoints^38^ and the associations between immune populations and response to immunotherapies^40^, these cell scores may hold information useful for monitoring or predicting responses to immunotherapy^39^. Cell scores were calculated as the average log2-normalized expression of each cell marker gene listed in Supplementary Table 1. The total tumor-infiltrating lymphocyte (TIL) score was calculated as the average of all cell scores whose correlations with PTRPC (CD45) exceeded 0.6. Cell-type enrichment scores were defined as the residuals of the regressions. These scores can be interpreted as a measurement of the abundance or depletion of each cell population relative to the total number of TILs.

### Immune microenvironment characterization

The scores of immune microenvironment characteristics generated using the gene expression data were calculated as the sum of the average log2-normalized expression of all marker genes for each characteristic. The following nine biomarkers were included in the analysis: adhesion molecules, chemokines, cytolytic activity, immunocostimulators, immunoinhibitors, MHC-class-I, MHC-class-II, non-class^41^, and IFNg-signature^42^. Gene sets of these signatures used are listed in Supplementary Table 2.

### Animal models

To further verify the results in vivo, we established an MPLC animal model through the implantation of concurrent ADC and SQCC in bilateral lungs of mice. Three wild-type C57BL/6 mice (purchased from Quicell, Shanghai, China) weighing 20–25 g at 6–8 weeks of age were anesthetized with isoflurane. Subsequently, the bilateral regions of the rib cage of the mice were shaved and sterilized. The skin was then incised 10 mm parallel to the ribs bilaterally, and the subcutaneous fat and muscular layer were dissected until the lungs were visible through the pleura. Then, KLN-205 cells (mice SQCC cell line, M0-0201, bought from Oricell, right lung, 2 × 10^6^/each mouse suspended in 10 μL of 1× phosphate-buffered saline [PBS]) mixed with 10 μL of Matrigel (#356234, Corning, Corning, NY, USA) and Lewis lung carcinoma (LLC) cells (mice ADC cell line, FH1074, bought from Fuheng Biology, left lung, 2 × 10^6^/each mouse suspended in 10 μL of 1× PBS) mixed with 10 μL of Matrigel were injected into the lungs bilaterally at a depth of approximately 2 mm through the pleura using a 30 G microinjector syringe (Shanghai Gaoge, 20 μL)^43^. After injection, the incisions were sutured layer by layer, and the mice resuscitated from anesthesia following intraperitoneal injection of penicillin (5 mg/each mouse) to prevent infection. On the 10th postoperative day, the mice were euthanized by cervical dislocation, and bilateral lung specimens were collected. Then, three pairs of concurrent ADC and SQCC lesions were selected and preserved in paraformaldehyde for fixation. All animal studies were conducted under a protocol approved by the Animal Ethics Committee of Shanghai Jiao Tong University School of Medicine.

### Multiplex immunohistochemistry (mIHC)

mIHC was conducted at Genecast Biotechnology Co., Ltd. (Beijing, China)^44^. Briefly, paraffin-embedded tumor tissues from both patients and mice were sectioned into 4-μm-thick slices. The slides underwent deparaffinization, rehydration, epitope retrieval, and protein blocking. Next, the slides were incubated with primary antibodies. Based on the transcriptomics data and the TIME panels, which had been extensively reported before^44^, we selected CD8, CD68, PANCK, PD-1, and PD-L1 as the mIHC indicators. The primary antibodies for human CD8 (ZA-0508, Zsbio, 1:100), CD68 (ZA-0060, Zsbio, 1:100), PANCK (ZM-0069, Zsbio, 1:100), PD-1 (ZM-0381, Zsbio, 1:50), and PD-L1 (ZA-0629, Zsbio, 1:50) were incubated with the human samples for 1 h at 37 °C, followed by incubation with a secondary antibody (PV-8000, Zsbio) for 10 min at 37 °C. At the same time, the primary antibodies against mouse CD8 (98941 T, CST, 1:100), CD68 (97778 S, CST, 1:100), PANCK (4545 T, CST, 1:100), PD-1 (84651 T, CST, 1:100), and PD-L1 (64988 T, CST, 1:50) were incubated with mice FFPE sections. Then, all the samples were incubated with the secondary antibody (PV-8000, Zsbio) for 10 min at 37 °C. Finally, the slides were incubated with opal (Akoya Biosciences) for tyramine signal amplification (TSA) visualization. The slides, including whole slices, were then scanned using the TissueFAXS SL (7.1.120) panoramic tissue cell imaging system. Tissue and cell-type identification and protein expression quantification of the scanned images were performed using the StrataQuest (7.1.129) analysis software.

### Statistical analysis

To compare two patient groups in terms of biomarkers (TNB, TMB, and MATH), the non-parametric paired Wilcoxon-rank sum test (two-sided) was used, and the paired *t* test was used for group comparisons of two biomarkers, including GSVA scores, immune cell scores, TIME biomarker scores, and cell density. *p* < 0.05 was considered to indicate statistical significance.

### Reporting summary

Further information on research design is available in the Nature Research Reporting Summary linked to this article.

### Supplementary information

supplemental metarials

REPORTING SUMMARY

## Data Availability

The sequencing data of 5 patients with MPLCs harboring concurrent ADC and SQCC in this article has been uploaded to the Genome Sequence Archive for Human (GSA-Human), following the Guidance of the MOST by the Human Genetic Resources Administration of China (HGRAC). The sequencing data and information of the research participants are not publicly available to prevent the disclosure of individuals’ genetic identities. Further analysis of sequencing data will be made available for collaborating researchers upon request, dependent on the HGRAC’s approval.

## Code availability

This study made use of publicly available packages with R version 4.2.0. There are no custom computer codes or scripts used to generate results reported in the manuscript. The code used to generate figures is available upon request.
