# Supplementary material for: Immune microenvironment heterogeneity of concurrent adenocarcinoma and squamous cell carcinoma in multiple primary lung cancers
Source: NPJ Precis Oncol. 2024 Feb 29;8:55. doi: 10.1038/s41698-024-00548-3 (PMC10904822; doi:10.1038/s41698-024-00548-3)
Supplement: Supplementary file 1 — supplemental metarials [file 41698_2024_548_MOESM1_ESM.pdf]

CNV landscape of adenocarcinoma and squamous cell carcinoma lesions

CNVs (copy number variants) refer to changes in the number of copies of a region on a chromosome. Supplementary Figure 1 shows the CNV landscape of ADC and SQCC patients. The genes with the most significant copy number changes in the ADC group were *EGFL7*, *ZNF98*, *GOLGA8CP*, *GOLGA8DP*, *GOLGA8F*, *GOLGA8G* and *ZNF99* (Supplementary Figure 1A, mutation frequency ≥ 80%), and *LMNTD2* (Supplementary Figure 1B, mutation frequency ≥ 80%) in SQCC group, respectively.

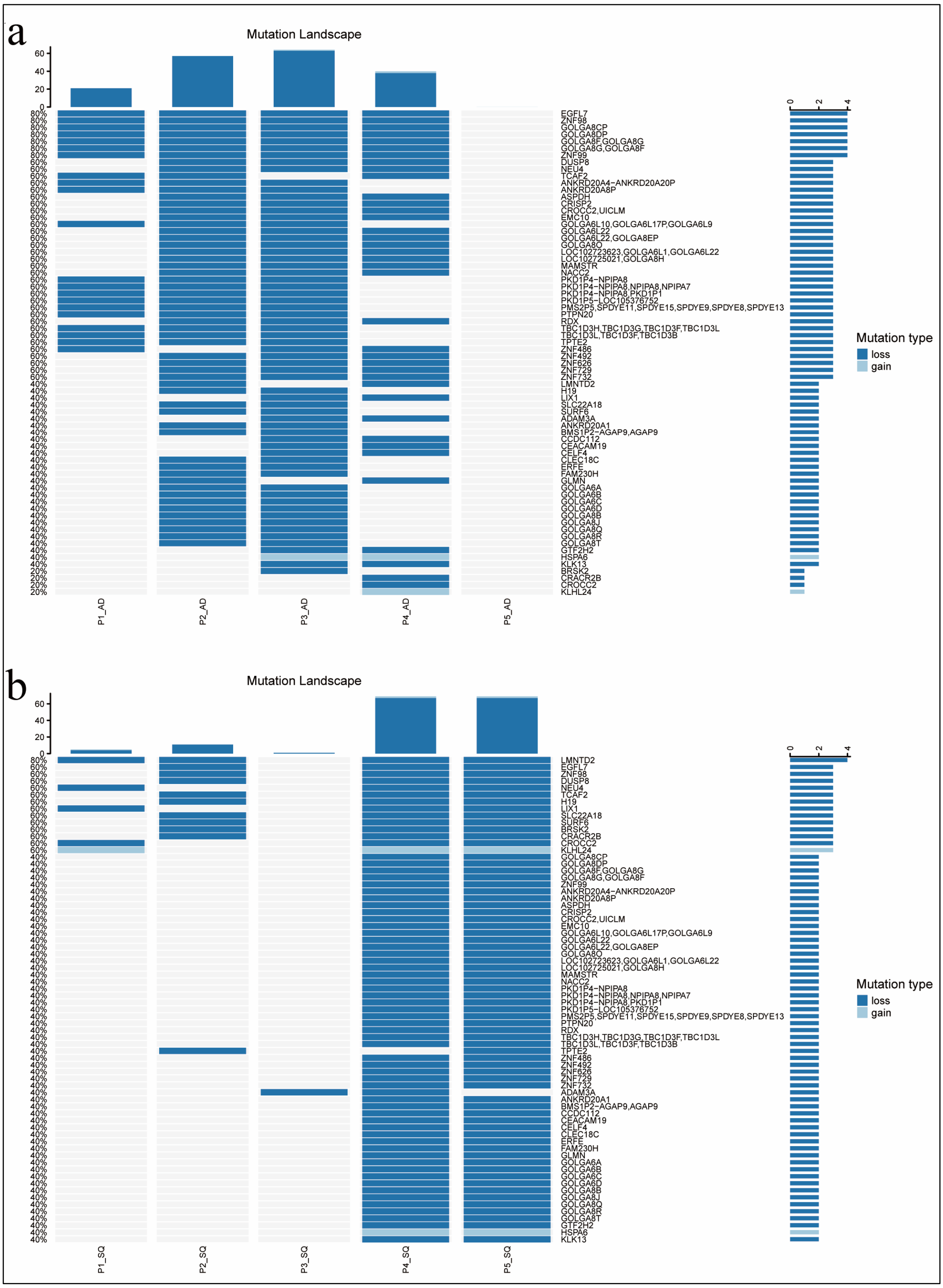

Supplementary Figure 1. CNV landscape of adenocarcinoma and squamous cell carcinoma lesions.

A, CNV landscape of 5 lesions in adenocarcinoma group; B CNV landscape of 5 lesions in squamous cell carcinoma group.

Exhausted CD8+ T cell-associated genes expression between MPLC

adenocarcinoma and squamous cell carcinoma lesions

In cancer, exhausted CD8+ is hyporesponsive to tumor cells[49]. We analyzed the expression of genes associated with exhausted CD8+ T cells with paired t-tests between SQCC and ADC lesions[50]. The analysis showed (Supplementary Figure 2A) that the overall trend of exhausted CD8+ T cell-related marker expression was more obvious in the ADC group; the analysis of specific group differences (Supplementary Figure 2B) showed that in the ADC group *CD244* ($p = 0.094$), *PTGER4* (Prostaglandin E 2 receptor 4) ($p = 0.338$), *PDCD1* (PD-1) ($p = 0.105$), *CD274* (PD-L1) ($p = 0.096$), *EOMES* (Eomesodermin) ($p = 0.108$), and *LAG3* (Lymphocyte activation gene 3) ($p = 0.161$) were higher than those in the SQCC group, but the differences were not significant ($p > 0.05$). This suggests that the abundance of exhausted CD8+ T cells and their depletion may be higher in ADC than in SQCC, thus ADC may be more likely to benefit from immune checkpoint inhibitor therapy.

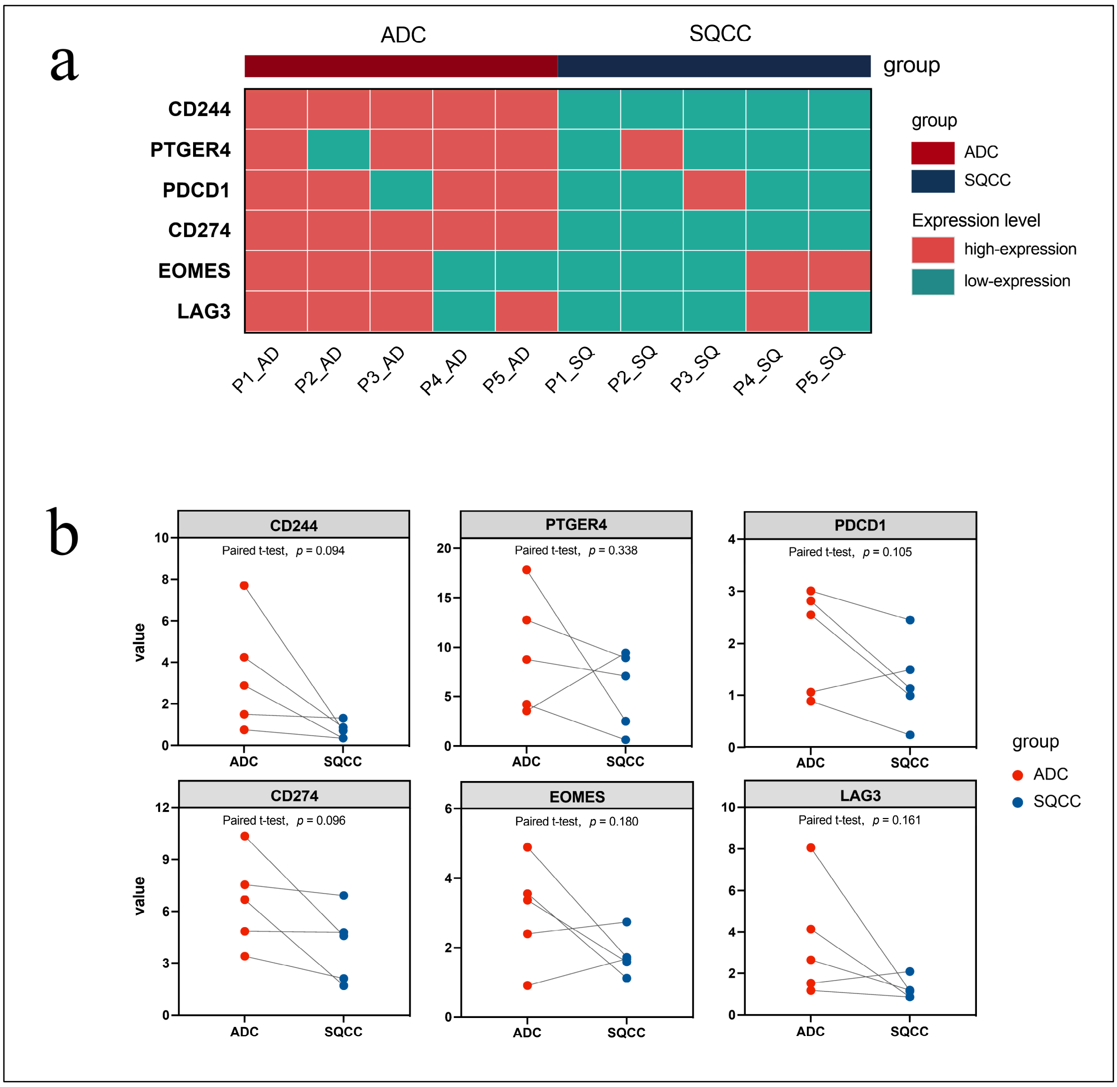

Supplementary Figure 2. Expression analysis of exhausted CD8+ T cell-associated genes between groups of MPLC adenocarcinoma samples and squamous carcinoma samples.

A. Double heat map of exhausted CD8+ T cell-associated genes expression between two groups. High-expression, the relative expression level of gene sets in one lesion higher than the histologically paired lesion (eg. P1_AD and P1_SQ). B. Paired scatter-link plot of CD244, PTGER4 (Prostaglandin E 2 receptor 4), PDCD1 (PD-1), CD274 (PD-L1), EOMES (Eomesodermin), and LAG3 (Lymphocyte activation gene 3) expression between two groups.

Gene	Cell Type
BLK	B-cells
CD19	
MS4A1	
TNFRSF17	
FCRL2	
KIAA0125	
PNOC	
SPIB	
TCL1A	
MS4A2	Mast cells
TPSAB1	
CPA3	
HDC	
TPSB2	

Gene	Cell Type
PTPRC	CD45
CD8A	CD8 T cells
CD8B	
CTSW	Cytotoxic cells
GNLY	
GZMA	
GZMB	
GZMH	
KLRB1	
KLRD1	
KLRK1	
PRF1	
NKG7	
NCR1	NK cells
XCL2	
XCL1	

Gene	Cell Type
CCL13	DC
CD209	
HSD11B1	
CD244	Exhausted CD8
EOMES	
LAG3	
PTGER4	
CD163	Macrophages
CD68	
CD84	
MS4A4A	
IL21R	NK CD56dim cells
KIR2DL3	
KIR3DL1	
KIR3DL2	

Gene	Cell Type
CSF3R	Neutrophils
S100A12	
CEACAM3	
FCAR	
FCGR3B	
FPR1	
SIGLEC5	
CD3D	T-cells
CD3E	
CD3G	
CD6	
SH2D1A	
TRAT1	
TBX21	Th1 cells
FOXP3	Treg

Supplementary Table 1. Cell marker gene lists used in Scoring of Tumor Infiltrating Lymphocytes. DC, dendritic cells; Treg, regulatory T cells; NK cells, natural killer cells

Gene	Signature name	Gene	Signature name	Gene	Signature name	Gene	Signature name
IDO1	IFNg-signature	HLA-DPA1	MHC-Class-II	CTLA4	Immunoinhibitors	CD27	Immunocostimulators
CXCL10		HLA-DPB1		PDCD1		CD28	
CXCL9		HLA-DPB2		LAG3		CD40LG	
HLA-DRA		HLA-DQA1		BTLA		CD40	
STAT1		HLA-DQB1		CD160		CD70	
IFNG		HLA-DQB2		IDO1		CD80	
B2M	MHC-Class-I	HLA-DRB1		IL10		CD86	
TAP1		HLA-E	Non-Class	TIGIT		ICOS	
TAP2		HLA-F		TGFB1		IL6	
HLA-A		HLA-G		HAVCR2		TMEM173	
HLA-B		CCR5	Chemokines	CD274		TNFRSF13B	
HLA-C		CCL3		PDCD1LG2		TNFRSF14	
ICAM1	Adhesion-molecules	CCL4		GZMA	Cytolytic-activity	TNFRSF17	
ICAM2		CCL5		PRF1		TNFRSF18	
ICAM3		CXCL9				TNFRSF4	
ICAM4		CXCL10				TNFRSF9	
ICAM5		CXCL11				TNFSF13B	
VCAM1						TNFSF13	

Supplementary Table 2. Lists of genes used in Immune microenvironment characterization.
